# Seed Meals from *Brassica nigra* and *Eruca sativa* Control Artificial *Nosema ceranae* Infections in *Apis mellifera*

**DOI:** 10.3390/microorganisms9050949

**Published:** 2021-04-28

**Authors:** Antonio Nanetti, Luisa Ugolini, Giovanni Cilia, Eleonora Pagnotta, Lorena Malaguti, Ilaria Cardaio, Roberto Matteo, Luca Lazzeri

**Affiliations:** 1 Research Centre for Agriculture and Environment (CREA-AA), Council for Agricultural Research and Agricultural Economics Analysis, Via di Saliceto 80, 40128 Bologna, Italy; antonio.nanetti@crea.gov.it (A.N.); ilaria.cardaio@crea.gov.it (I.C.); 2 Research Centre for Cereal and Industrial Crops (CREA-CI), Council for Agricultural Research and Agricultural Economics Analysis, Via di Corticella 133, 40128 Bologna, Italy; luisa.ugolini@crea.gov.it (L.U.); eleonora.pagnotta@crea.gov.it (E.P.); lorena.malaguti@crea.gov.it (L.M.); roberto.matteo@crea.gov.it (R.M.); luca.lazzeri@crea.gov.it (L.L.)

**Keywords:** *Nosema ceranae*, *Apis mellifera*, IPM, Brassicaceae defatted seed meal, glucosinolates, isothiocyanate

## Abstract

*Nosema ceranae* is a widespread parasite responsible for nosemosis Type C in *Apis mellifera* honey bees, reducing colony survival. The antibiotic fumagillin is the only commercial treatment available, but concerns are emerging about its persistence, safety, and pathogen resistance. The use of natural substances from Brassicaceae defatted seed meals (DSMs) with known antimicrobial and antioxidant properties was explored. Artificially infected bees were fed for 8 days with candies enriched with two concentrations, 2% and 4%, of two DSMs from *Brassica nigra* and *Eruca sativa*, containing a known amount of different glucosinolates (GSLs). The food palatability, GSL intake, bee survival, and treatment effects on *N. ceranae* spore counts were evaluated. Food consumption was higher for the two 2% DSM patties, for both *B. nigra* and *E. sativa*, but the GSL intake did not increase by increasing DSM to 4%, due to the resulting lower palatability. The 2% *B. nigra* patty decreased the bee mortality, while the higher concentration had a toxic effect. The *N. ceranae* control was significant for all formulates with respect to the untreated control (312,192.6 +/− 14,443.4 s.e.), and was higher for 4% *B.* nigra (120,366.3 +/− 13,307.1 s.e.). GSL hydrolysis products, the isothiocyanates, were detected and quantified in bee gut tissues. Brassicaceae DSMs showed promising results for their nutraceutical and protective effects on bees artificially infected with *N. ceranae* spores at the laboratory level. Trials in the field should confirm these results.

## 1. Introduction

*Nosema ceranae* is a honey bee pathogen that belongs to the Microsporidia class [1]. Like other microsporidia, the *N. ceranae* is an obligate intracellular parasite able to colonize the midgut epithelium of *Apis* spp. [1,2]. The parasite is generally considered associated with the Asian honey bee *Apis ceranae* [3], but in the early 2000s, *N. ceranae* rapidly spread in the western honey bee *Apis mellifera,* causing nosemosis Type C [4,5,6]. The *N. ceranae* infection causes damage at the individual and colony level due to its increased energetic stress, reducing the lifespan, downregulating the genes involved in intestinal health and the absorption of nutritional compounds, inducing lethargic behavior with poor honey and pollen harvest, and suppressing the immune response, downregulating the expression of antimicrobial peptides (AMPs) [7,8,9,10,11,12,13]. All of these factors are involved in the decline and collapse of honey bee colonies [7,14]. The only effective medicament against nosemosis is fumagillin, an antibiotic mycotoxin isolated from *Aspergillus fumigatus*, used for over 60 years in the beekeeping industry [15,16]. Although fumagillin may reduce the *N. ceranae* infection, its use is not legal in several countries as it may build residues in the bee products and result in antibiotic resistance; thus, promoting parasite proliferation [17]. Recently, natural and synthetic compounds were tested as a treatment against nosemosis Type C [18,19,20,21,22,23]. A commercial dietary supplement based on herbs (garlic and cinnamon) and vitamins, the ApiHerb^®^ (Chemicals Laif S.p.A., Padua, Italy) showed a significant effect against *N. ceranae* infections [17,24,25] on the microbial profile of the honey bee midgut [26,27]. Moreover, the oxalic acid, which is generally administered to the colonies as a means to control *Varroa destructor* infestations, likewise showed efficacy against *N. ceranae* infections [17,28,29]. Moreover, sugars and protein administrated in sucrose syrup were effective to reduce *N. ceranae* infection in bees [30]. In detail, chitosan and peptidoglycan significantly reduced the nosemosis infection level in infected bees, and significantly increased their survivorship [30]. Even prebiotic and probiotic administration could be useful to reduce *N. ceranae* [31,32]. The administration of two probiotics, Protexin Concentrate single-strain (*Enterococcus faecium*) and Protexin Concentrate multi-strain (*Lactobacillus acidophilus, L. plantarum, L. rhamnosus, L. delbrueckii, Bifidobacterium bifidum, Streptococcus salivarius,* and *E. faecium*) significantly reduce the number of spores in infected bees without affecting their surviving [31]. Moreover, the probiotic effect of two bee gut bacteria (*Parasaccharibacter apium* and *Bacillus* sp.) and two commercial probiotics (Bactocell^®^ and Levucell SB^®^) increase the survival in *N. ceranae* infected honey bees [32].

**Figure 1:**
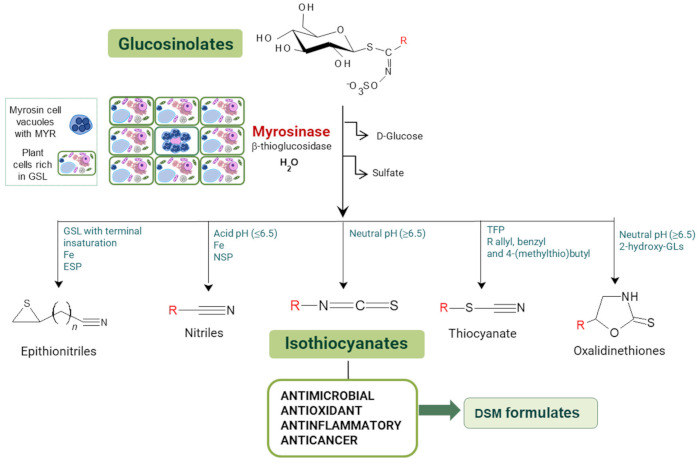
General pathway of myrosinase (MYR)-catalyzed hydrolysis of glucosinolates (GSLs). TFP, thiocyanate-forming protein; ESP, epithiospecifier protein; NSP, nitrile-specifier protein; DSM defatted seed meal.

Among the plants known to contain bioactive compounds with antimicrobial and antioxidant activity, the Brassicaceae family includes species that historically belong to the human diet, and are widely cultivated as important oilseed crops. Vegetable oils, in particular those from Brassica oilseeds, are well known for their potential applications in green chemistry, including their use as hydraulic fluids, biolubricants, and cosmetics [33]. Biomolecules produced from Brassica oilseed co-products, namely defatted seed meals (DSMs), can be applied in food and feed industries and as sustainable tools for agriculture, thus representing an innovative solution for the agricultural and processing phases [34,35,36,37]. Brassicaceae are characterized by the presence of the endogenous defense mechanism against pathogen attacks, the glucosinolate–myrosinase (GSL–MYR) system [38,39]. GSLs are plant secondary metabolites structured by a common hydrophilic β-d-glucopyranose residue linked via a sulfur atom to a (Z)-*N*-hydroximinosulfate ester, plus a variable hydrophobic aglycone side chain (R-group) derived from amino acids (Figure 1).

More than 130 GSL structures have been discovered and validated to date, and they may be classified in aliphatic, aryl aliphatic, or indolic GSLs in function of the variable side chain structure [40,41]. A single GSL is often dominant in brassica plants and their GSL quali–quantitative composition largely depends on the plant genotype, growth stage, the cultivation conditions, and the plant organ, even if GSLs are often particularly concentrated in seeds [42]. GSLs consistently occur in plants, in conjunction with the hydrolytic enzyme ¦Â-thioglucosidase, MYR, (E.C. 3.2.1.147). Upon biotic or abiotic lesions and physical disruption of the plant cell, GSL and MYR come into contact, triggering, in the presence of water, the hydrolysis reaction that releases a broad range of degradation compounds [43]. The isothiocyanates (ITCs) are the main products formed at neutral pH, but other compounds could form in function of several factors as substrate structure, reaction conditions, and the presence of specifier proteins (Figure 1).

ITCs are bioactive compounds largely known for their broad-spectrum biological activity against pests and soil/food-borne fungi and bacteria [44,45,46,47,48,49]. They are also studied for their beneficial effects in human health, chemo-protective, and antioxidant activities, assayed in vitro, in vivo, in human and in epidemiological studies [50]. Sulforaphane, mainly present in broccoli and black kale sprouts, is the most frequently studied ITC for its preventive activity against a variety of cancers, cardiovascular, neurodegenerative diseases, and diabetes [51], but also showed inhibitory activity against bacteria and fungi through in vitro trials [52]. Moreover, ITCs exert their health effect through antimicrobic activity against common human pathogens, such as *Helicobacter pylori* [53,54]. More recently, broths of lactic acid bacteria fermented with *Eruca sativa* seed extracts showed promising results in preventing *Escherichia coli*-induced intestinal inflammation and barrier dysfunction in Caco-2 cell in vitro assays [55]. Furthermore, Brassicaceae vegetables are a rich source of other health-promoting nutrients and phytochemicals, apart from GSL, such as vitamins (ascorbic acid, carotenoids), minerals, fibers, soluble sugars, amino acids, indole phytoalexins, terpenes, phytosteroids, and phenolic compounds, which contribute to their antioxidant and anti-inflammatory protective effects [56]. Although GSL derives product activity against plant pathogens and pests, and its beneficial effects on human health are well documented and still under study, its potential protective role against animals, in particular insects and infections, has been less studied.

The present work aimed to explore the use of brassica DSMs in beekeeping to control *N. ceranae* infection. Artificially infected *A. mellifera* workers were reared and fed with different *B. nigra* and *E. sativa* GSL-containing diets to measure: (i) palatability and GLS intake; (ii) honey bee survival; (iii) development of the *N. ceranae* infection; and (iv) GSL conversion into ITCs in the honey bee intestines.

## 2. Materials and Methods

### 2.1. Source of Honey Bees

The experiments were conducted in the summer of 2018 on worker bees from an *A. mellifera ligustica* colony, selected from an apiary of CREA-AA located in Bologna, Italy (44°31′27.1″ N 11°21′03.6″ E); it was in appropriate conditions of development, and missing clinical symptoms of the disease. Combs with mature broods were taken from the nest and stored in an incubator at 34 °C. Hatching bees were collected daily in batches, incubated at 33 °C, fed ad libitum with sugar water (1:1 *w/w*), and supplied with water until the moment of the inoculation with *N. ceranae* spores.

### 2.2. Nosema ceranae Spore Extraction

An apiary with *A. mellifera ligustica* colonies was selected from the same area to provide *N. ceranae* spores. This was known as an infected apiary based on previous observations. Foragers were collected from the flight entrance of colonies showing signs of dwindling and kept at 33 °C under ad libitum feeding with sugar water until the extraction of the *N. ceranae* spores. The day before, or the same day, of each artificial infection session, 100–200 foragers were taken from the incubator and sacrificed. After dissection, their abdomens were smashed in a stomacher with a small amount of distilled water. The resulting material was filtered with a nylon mesh, washed with water to obtain approximately 20 mL suspension, and split into four aliquots. Each aliquot was gently overlaid onto a column of 40 mL 95% isotonic Percoll^®^ (Sigma-Aldrich, Saint Louis, MO, USA) in a 50 mL centrifuge tube. After repeated centrifugation and washing with PBS 1×, a pellet of purified spores was obtained and resolved in 250 µL PBS 1. The suspension was kept at room temperature until its use.

### 2.3. Brassicaceae DSMs

*Brassica nigra* (L.) W.D.J. Koch and *Eruca sativa* Mill. seeds were available in the seed collection of Brassicaceae of CREA-CI (Bologna) [57]. The two crops were cultivated at the experimental station of CREA-CI located at Anzola (Bologna), at open field level adopting low input techniques for both energy and fertilizers, whilst no pesticides were applied. Seeds were harvested, cleaned, and defatted using a small continuous seed crusher machine (Company model Elle.Gi type 0.90, Bracco, Milan, Italy) in a temperature-controlled procedure during which temperature was maintained at a maximum of 70 °C [58]. Obtained pellets were subsequently milled in a coffee grinder and the two defatted seed meals (DSMs) were treated in an autoclave, at 120 °C for 20 min for MYR enzyme deactivation. Finally, *B. nigra* and *E. sativa* deactivated DSMs were ground again by an ultra-centrifugal mill ZM200 (Retsch GMBH, Haan, Germany) and sieved at 80 μm to obtain a homogenous fine powder that was stored at room temperature in sealed vials until use. MYR deactivation was verified by suspending DSMs with 50 mM K phosphate buffer, pH 6.5 (1:10 *w/v*) for 20 min and 24 h, under agitation at room temperature, and verifying the DSM residual GSL content (GSL analysis described below). Obtained DSMs were characterized for moisture, nitrogen, and residual oil content according to [59]. The oil content was determined by the standard automated continuous extraction, following the Twisselmann principle, by using an E-816 ECE (Economic Continuous Extraction) extraction unit (BÜCHI Labortechnik AG, Flawil, Switzerland), and hexane as solvent. Nitrogen content was determined by using the Elemental analyzer LECO CHN TruSpec (St. Joseph, MI, USA) and the protein content was calculated from nitrogen using the conventional factor of 6.25. Oil and nitrogen content were expressed as a percentage on dry matter (DM). Oil fatty acid composition was analyzed by trans-methylation in 2N KOH methanol solution and fatty acid methyl esters (FAMEs) were evaluated by a gas chromatography-FID detector (HRGC 5300 MEGA SERIES Carlo Erba, Milan, Italy) by a capillary column Restek RT × 2330 (30 m × 0.25 mm × 0.2 µm). Total GSL content and profile were determined by HPLC analysis of desulfo-GSLs following the ISO 9167-1 method [60], with some minor modifications [61], by using a Hewlett-Packard 1100 HPLC (Agilent Technologies, Santa Clara, CA, USA) equipped with a diode array detector and an Inertsil 5 ODS 3 column (250 × 3 mm). The desulfo-GSLs were detected monitoring their absorbance at 229 nm and identified by UV spectra and HPLC retention times according to a purified standard library. Their amount was estimated using sinigrin (2-propenyl GSL–SIN) and epi-progoitrin ((R)-2-hydroxybut-3-enyl GSL–EPI) as internal standards. The response factors used for desulfated glucoerucin (4-methylthiobutyl GSL–GER) and glucoraphanin (4-methylsulfinylbutyl GSL–GRA) determination in *E. sativa* were chosen according to [62], while for the desulfated SIN in *B. nigra*, the response factor was experimentally determined as 0.92. SIN and EPI standards were isolated from *Brassica juncea* and *Crambe abyssinica* Hochst. ex R. E. Fries seeds, respectively, according to [63], with a purity of 98.7% for SIN and 92% for EPI, as indicated by HPLC–UV chromatograms, and >96% on a weight basis for both GSLs. Glucoerucin (4-methylthiobutyl GSL–GER) and GRA reference standards were purified from *E. sativa* Mill and *Brassica oleracea* L. var. *acephala sabellica,* respectively, yielding for both GSLs, 99% purity based on the HPLC peak area value and 96% purity on weight-based. Total phenolic content was determined by ultrasound-assisted extraction of the DSM with acidified aqueous methanolic solutions and the Folin–Ciocalteu method using an Infinite M200 PRO microplate reader (Tecan, Männedorf, Switzerland) according to [48]. Gallic acid was used as standard, and the results were expressed as mg gallic acid equivalent (GAE)/g of DSM.

### 2.4. Feed Formulation

Deactivated DSMs of *B. nigra* and *E. sativa* were mixed at two different concentrations, 2% and 4% (*w/w*), with sugar candy ApiCandy (Chemicals Laif, Padua, Italy) and 1% of water by an electric mixer. The sugar candy was composed of water at 3%; pH 5.5; carbohydrates at 97% (fructose 7.5%, glucose 8%, and sucrose >60%). The components were mixed until homogeneity, divided in aliquots of about 9 g per treatment cage, placed in empty plastic syringes (5 mL), and weighted. Syringes were previously cut at the edge to facilitate bee feeding during trials. GSL content stability of DSM patties, stored at 33 °C in ended-cut syringes, was verified at the beginning and the end of trials, after hot ethanol-water (70%) extraction and GSL analysis performed as described above (Section 2.3). A candy added with water 1% was prepared following the same protocol (negative control, CTRL−).

### 2.5. Experimental Design

#### 2.5.1. Artificial Infections and Uninfected Groups

Before bee infection, the *N. ceranae* spore concentration of the isolated suspension was assessed with a Burker hemocytometer, then an aliquot was mixed with sugar water to the final concentration of 75,000 spores/µL. Five-day-old bees taken from the incubator were starved for approximately 2 h and gently transferred into Petri dishes in batches of five. The bee manipulation was eased by mild narcotization with a mixture of air and CO_2_ (40/60%, in volume). The bees were individually inoculated by feeding 2.5 µL purified spore suspension with a micropipette, corresponding to 187,500 spores/bee. The distribution of the experimental errors between the groups was accomplished by introducing the five inoculated bees of each batch randomly into hoarding cages (polystyrene, approximately 10 × 11 × 5.5 cm, with transparent walls and holed roof to supply food and water) until the needed number (N = 25). Two syringes filled respectively with sugar water and water were introduced from the holed roof into each cage for ad libitum feeding and watering. The cages were kept in an incubator at 33 °C for one day, to let the bees recover from the manipulation stress before the treatment.

The procedure described above was used also to arrange cages with uninfected five-day-old bees, which were administered 2.5 µL sugar water per os with a micropipette and kept in a separate incubator at 33 °C to prevent cross infections. The treatments were made after ad libitum administration of sugar water and water, for one day, as described for infected bees.

#### 2.5.2. Bee Treatment Feeding Trials

The experiment on infected bees aimed to measure the effect on bee palatability, tolerability, and *N. ceranae* infection of DSM patties from *B. nigra* (N) and *E. sativa* (R), prepared as described above (Section 2.4). Treatments started one-day post-infection (dpi) and included four treated groups fed with DSM patties enriched with two doses of Brassicaceae DSM, 2 and 4%, both for *B. nigra* and for *E. sativa* (N2, N4, R2, R4), and two controls each receiving unadded candy (CTRL−) or sugar water added with 25 µg/mL fumagillin (CTRL+). The six cages containing the infected bees (N = 25) were randomly assigned to the groups and incubated at 33 °C throughout the trial. Three replicates were made for each treatment. Solid and liquid feeds were supplied *ad libitum* and integrated with fresh food on the need. Unlimited water availability was secured by a syringe until the end of the trial.

DSM patty palatability was performed at a 1–3-day interval after inoculation by weighting the feeders and calculating the food consumption by the difference of weight. The calculated amount was divided by the number of days to obtain the daily consumption, and by the number of bees that were present in the cage at the beginning of the interval, to calculate the average individual daily intake. This amount, calculated a daily basis for each cage, was summed up for the first seven days of the treatments (8 dpi) to calculate the weekly cumulative individual ingestion. Multiplying the food ingestion data by the respective GSL concentration allowed calculating the average individual GSL intake that occurred in each cage. Ingestion data of the CTRL+ were not reported as this group received syrup instead of solid food.

The cages were inspected at 1–3-day intervals to determine even bee survival, by counting the dead bees. After counting, the dead bees were removed from the cages.

Living bees were sampled from each cage the 6, 8, 10, 13, and 15 dpi, and frozen at −20 °C until the molecular analysis was performed (Section 2.6). Whenever possible, five bees were collected per cage, even if the bee mortality prevented a complete sampling plan in all groups.

During the inspections, three and five bees escaped, respectively, from the cages with infected and uninfected groups. Those individuals were removed from the dataset and further ignored.

### 2.6. N. ceranae Infection Quantification: DNA Extraction and qPCR

Before DNA extraction, 1ml of DNA-free water was added to the individual bee samples. Homogenates were obtained by a Tissue Lyser II (Qiagen, Hilden, Germany) running at 30 Hz for 3 min, as previously described [46]. The total DNA was extracted from each resulting homogenate with a Quick DNA Microprep Plus Kit (Zymo Research, Irvine, CA, USA) following the modified protocol for solid tissues [17]. The Real-Time qPCR to quantify the number of genomic copies of *N. ceranae* was performed using primers and probes designed on sequences of the *Hsp70* gene [64]. A total reaction volume of 15 µL was prepared with 2× QuantiTect Probe PCR Master Mix (Qiagen, Hilden, Germany), forward and reverse primers (2 µM), probe (500 nM), and 3 µL DNA extract. A standard curve was obtained by amplifying the *N. ceranae*-specific DNA fragment diluted serially from 1 × 10^0^ to 1 × 10^9^ copies as previously described [17].

The Real-Time qPCR assays were performed on a Rotor-Gene Corbett 6000 (Corbett Research, Sydney, Australia) following the amplification and quantitation protocols for the gene sequence [64]. All the analyses were conducted on two technical replicates.

### 2.7. Bee GSL Metabolism Investigation

#### 2.7.1. Bee Treatment and Gut Extraction

Cages with uninfected bees were prepared as described above for a study on the release within the honey bee intestines of hydrolysis products from the GSLs present in the DSM patties. The trial consisted of three replicates starting the same days as the previous experiment. Each of them included two groups treated with DSM patties N2 and R2 and one CTRL− receiving unadded candy. In this case, the groups were randomly assigned to the treatments and received ad libitum feeding and water supply from one dpi. On day 7 post-treatment, the surviving bees were sacrificed and dissected. On a Petri dish placed on an ice block, their midgut and hindgut were separated and grouped (N = 5) into different 1.5 mL tubes, which were promptly deep-frozen by immersion into liquid nitrogen and stored at −80 °C until analysis. By this, depending on the number of surviving bees, it was possible to obtain 3–5 technical replicates from each cage.

#### 2.7.2. Total ITC Gut Analysis

Sample guts were extracted two times with 300 μL and 250 μL of cold pure methanol using a Tissue-Lyser II (Qiagen, Hilden, Germany) (30 Hz, 5 × 30 s), and subsequently centrifuged at 31,500× *g* for 20 min at 4 °C. A combination of the extracts (500 μL) was used for the cyclocondensation assay with 1,2-benzenedithiol to quantify the total ITCs as described in [48]. The cyclocondensation product, the 1,3-benzodithiole-2-thione, was analyzed using a Hewlett-Packard chromatograph 1100 (Agilent Technologies, Santa Clara, CA, USA), equipped with a diode array detector and an Eclipse XDB-C8 HPLC column (150 × 4.6 mm, 5 μm; Hewlett Packard), thermostated at 30 °C. An external calibration curve was generated using methanolic solutions of pure allyl-ITC, AITC (Sigma-Aldrich, Saint Louis, MO, USA) and 4-methylthiobutyl ITC, erucin, to obtain the cyclocondensation product for ITC quantification in gut deriving from bees fed on N2 and R2, respectively. Results were expressed as pmol/mg of ITC in gut tissues. Erucin standard was produced and purified from the GER–GSL as described in [63] with a purity >98% (*w/w*).

### 2.8. Calculations and Statistics

The weekly cumulative food ingestion of the individual bees belonging to the treated groups and the CTRL were normally distributed and they were statistically analyzed by a principal effect ANOVA, using the kind of food (CTRL−, R, N) and the concentration (0, 2, 4%) as categorical predictors. After the F test, significant differences between the factor levels were investigated by a pairwise Newman–Keuls post-hoc test. The cumulative GSL intake at 8 dpi was statistically analyzed by the same procedure. However, in this case, the CTRL−group was excluded from the analysis.

The survival of infected bees was analyzed with the Kaplan–Meier product limit estimator. The individuals sampled from the cages to assess the infection level were considered as right-censored observations. The sampling activity implied an increasing number of censored cases, which limited the time available for the survival estimate. A log-rank/Mantel–Cox post-hoc test was conducted for pairwise comparison of the survival distribution between the six groups, under the null hypothesis of equality.

The instantaneous risk of death was calculated with the Cox proportional hazard regression model and reported as the exponential function of the regression coefficients (hazard ratio: HR). In this analysis, the CTRL− acted as the reference group and a Wald test was conducted to spot significant differences between the reference and each of the other groups.

The *N. ceranae* abundance of each sampled bee was determined as the average number of copies detected in the two technical replicates. A general linear model (GLM) was used to analyze this dependent variable against ‘treatment’ and ‘dpi’, respectively, as a categorical and continuous predictor. Sigma-restricted parametrization was used for the categorical predictor, and a between-group design in which the treatment levels were tested versus the CTRL+ was used. Thereafter, a Newman–Keuls pairwise post-hoc test allowed to evaluate the significance of the differences between the treatment levels.

GSL stability in DSM patties and gut ITC analysis results were subjected to a one-way analysis of variance employing the least significant difference (LSD) test to assess significant differences between the analyzed samples.

A protection level against the statistical type I error of p≤α=0.05 was set for all tests.

## 3. Results

### 3.1. DSM Characterization

Oil seed meal extraction was performed by a mechanical food-grade process, avoided high temperature, and use of solvents, which could impair the bee safety and the DSM quality too. *E. Sativa* and *B. nigra* DSM chemical characterization results are shown in Table 1.

*E. sativa* and *B. nigra* DSMs had a relatively high protein content, 35% and 43.8%, respectively, calculated from the DSM nitrogen quantification. A low percentage of residual oil was also present, higher for *E. sativa* than *B. nigra*, as a consequence of the defatting process. Both DSM oils were mainly composed of the long-chain erucic acid (22:1), 46% and 38%, respectively, plus around 10% of oleic acid (18:1) and 8% of gadoleic acid (20:1). A relatively high percentage, between 10% and 15%, of poly-unsaturated fatty acid, linoleic (18:2—omega-6) and linolenic acid (18:3—omega-3), were also present, higher in *B. nigra* than in *E. sativa* DSM.

The quali-quantitative GSL content of DSMs was also evaluated. The main GSLs in *E. sativa* belong to the class of aliphatic thio-functionalized GSLs, with a 4-carbon chain length, containing a sulfur atom at different oxidation states in the two GSL, with a total GSL content of 92.2 ¦μmol/g. The prevalent form is the GER, representing the 82% of total GSL, while the oxidized form GRA was present in a lower percentage. On the other hand, *B. nigra* DSM contains one prevalent GSL (>95%), the aliphatic short side chain SIN. GSLs are hydrolyzed by MYR at neutral pH to the corresponding ITCs, which maintain the side chain (R) structure of native GSL linked to the ITC (S=C=N-) moiety (Figure 1). Erucin and sulforaphane ITCs are the hydrolysis products of GER and GRA, respectively, and AITC of SIN. The total phenolic content was also quantified as DSM phytonutrient with antioxidant properties and no significant difference was found between the two DSMs.

### 3.2. DSM Formulation and GSL Stability in the DSM Patties

DSM patties were formulated for the treatment trials considering two DSM concentrations (2% and 4%) chosen based on preliminary trials evaluating palatability and tolerability (results not reported). GSL stability in the 4% DSM patties (N4 and R4) stored for 10 days at the same experimental conditions as in bee treatment feeding trials—that is, in syringes with a cut end, at 33 °C, was established. GSL patty content was determined at the beginning and the end of trials to observe the GSL stability over time; results are presented in Table 2.

SIN concentration in N4 was stable and no significant differences in its content were found in the residual patty before and after storage. Otherwise, the GER and GRA content showed small but significant changes, as the final GER concentration was 5.5% lower, while GRA was 7.6% higher than starting values.

### 3.3. Bee Treatment Feeding Trials

Bees were inoculated with isolated *N. ceranae* spores and treatment with DSM patties (N2, N4, R2, R4) started one dpi. Palatability, tolerability of DSM patties and their effect on *N. ceranae* infection were determined during trials and results are reported as follows.

#### 3.3.1. Palatability of DSM Patties and GSL Intake

**Figure 2:**
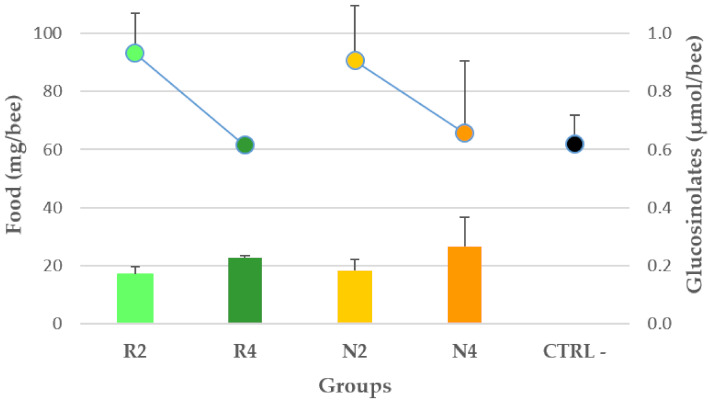
Cumulative individual food daily consumption of *N. ceranae*-infected treated groups (R2, R4, N2, N4) and CTRL−, expressed as mg/bee (dots—left *y*-axis), and relative cumulative individual GSL intake, expressed as ¦μmol/bees (columns–right *y*-axis), at 8 days post-infection. Vertical bars indicate the standard deviation.

Bee food consumption was established for the treatment groups N2, N4, R2, R4 patties and the CTRL− and calculated as cumulative individual daily consumption at 8 dpi. The cumulative DSM patty individual consumption was then converted into GSL individual consumption based on the DSM patties GSL content. For R groups the *E. sativa* DSM total GSL content, as the sum of GRE and GRA, was considered. The average cumulative intake was 74.6 +/− 5.2 s.e. (SD = 20.0, 95% CI = 63.5, 85.6) mg/bee corresponding to 0.213 +/− 0.018 s.e. (SD = 0.061, 95% CI = 0.174, 0.252) GSL µmol/bee. Figure 2 shows the food consumption and relative GSL intake that occurred in the three replicates of each group.

The kind of food did not significantly influence the individual food consumption (F (1, 11) = 0.010, *p* = 0.922), which instead significantly varied with the DSM concentration (F (1, 11) = 10.377, *p* = 0.008). Indeed, a post-hoc test showed a significantly higher average consumption of food added with 2% DSM compared to 4% and the CTRL−.

The individual GSL intake at 8 dpi did not significantly change with the kind of food (F (1, 9) = 0.772, *p* = 0.402) and the DSM patty concentration (F (1, 9) = 5.031, *p* = 0.051).

#### 3.3.2. Effect of DSM Patties on Bee Survival

**Figure 3:**
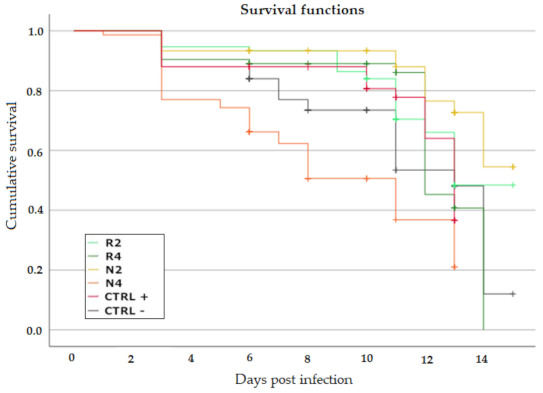
Kaplan–Meier survivorship curves for the *N. ceranae*-infected treated groups (R2, R4, N2, N4), CTRL+ and CTRL−. Crosses indicate right-censored observations (cens.).

On average, the infected bees (N = 447, including 306 censored cases) survived 11.682 +/− 0.209 (s.e.) dpi. Figure 3 and Table 3 report, respectively, the Kaplan–Meier curves and the mean survival for the individual groups.

The pairwise log-rank/Mantel–Cox test did not show a significant difference between CTRL− and CTRL+ (¦χ^2^ = 1.018, *p* = 0.313).

Bees in group N2 lived significantly longer than both CTRL− (¦χ^2^ = 11.870, *p* = 0.001) and CTRL+ (¦χ^2^ = 6.335, *p* = 0.012), and the treated bees of group R4 (¦χ^2^ = 5.540, *p* = 0.019). Bees receiving N4 survived significantly less than any other group: CTRL− (¦χ^2^ = 8.418, *p* = 0.004) and CTRL+ (¦χ^2^ = 14.721, *p* = 0.000), N2 (χ^2^ = 33.371, *p* = 0.000), R2 (χ^2^ = 21.353, *p* = 0.000) and R4 (χ^2^ = 18.044, *p* = 0.000). Therefore, results of N feeding effect on bee survival are strongly affected by the DSM concentration, with the high and low concentration having opposite consequence on bee. The same did not occur with R, as no significant difference was recorded between the two tested concentrations (χ^2^ = 0.121, *p* = 0.728). Moreover, they indicate lower compatibility with the bees of patties formulated with N4 vs. R4. However, the bee survival was not significantly affected by the lower DSM concentration in N2 and R2 groups (χ^2^ = 3.182, *p* = 0.074).

**Figure 4:**
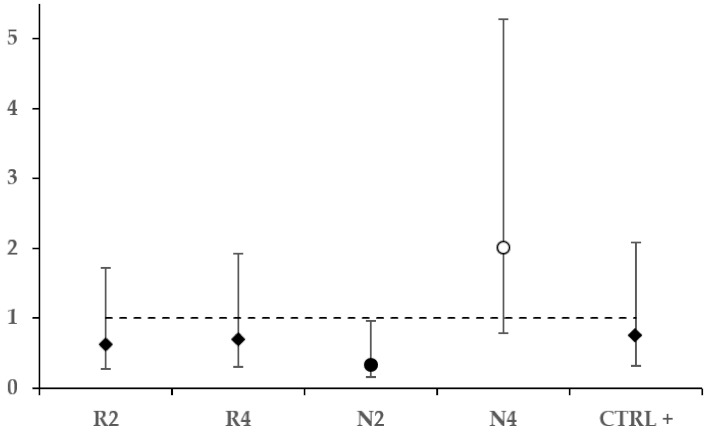
Hazard ratios represented along with the respective confidence interval for each experimental *N. ceranae*-infected treated group (R2, R4, N2, N4) and CTRL+. The CTRL− served as the reference group. The dotted line indicates HR = 1. Significantly lower, higher, and non-significant HR values are, respectively, indicated by a black circle, white circle, and diamonds.

The Cox proportional hazard regression model (Figure 4) indicates the kind of food as a significant predictor of bee survival (χ^2^ = 40.472, *p* = 0.000).

Significant HR = 0.322 (χ^2^ = 10.698, *p* = 0.001) and HR = 2.000 (χ^2^ = 7.468, *p* = 0.006) were calculated, respectively, for the groups N2 and N4.

#### 3.3.3. Effect of DSM Patties on *N. ceranae*

**Figure 5:**
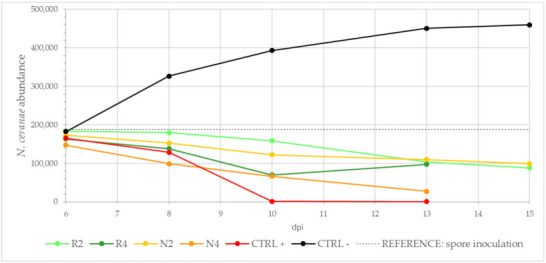
Variation in the *N. ceranae* copies across the timeline in the different treated groups (R2, R4, N2, N4), CTRL+ and CTRL−.

Figure 5 shows the number of *N. ceranae* copies detected with the RT-PCR in the different groups during the experiment.

The GLM approach including ‘*N. ceranae* abundance’ as the univariate dependent variable and ‘treatment’ and ‘dpi’ as independent predictors resulted in a significant model (R^2^ = 0.529, F (6, 299) = 56.082, *p* = 0.000). The relatively low coefficient of determination accounts for both the individual data variability and the non-linear component, which is rather evident in some of the groups. Within this model, highly significant predictors were both the independent continuous (dpi: F (1, 299) = 19.267, *p* = 0.000) and categorical (treatment: F (5, 299) = 62.880, *p* = 0.000) variable.

A significant model intercept was calculated, with a value (216,340.0) reasonably close to the spore dose (187,500) that was used to artificially infect the bees (Table 4).

**Figure 6:**
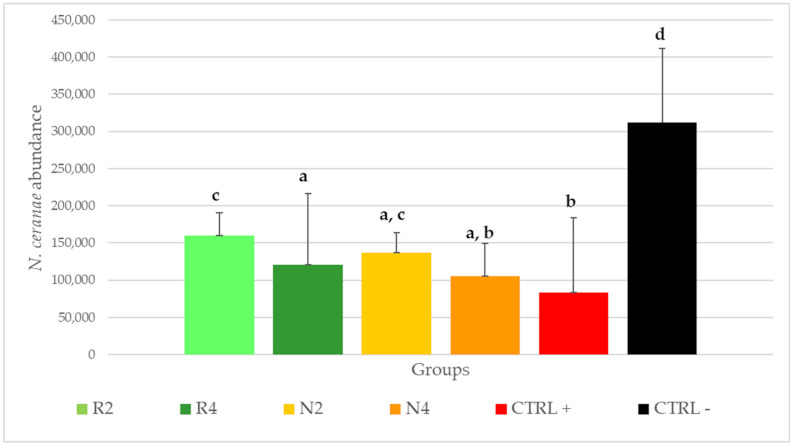
Average *N. ceranae* abundance copies calculated for the different levels of the factor ‘treatment’, for treated groups (R2, R4, N2, N4), CTRL+ and CTRL−. Standard deviations are shown as vertical bars. Same letters indicate no significant differences (Newman-Keuls post-hoc test; *p* = 0.05).

The average infection level of individual bees belonging to the different groups is reported in Figure 6.

The number of *N. ceranae* copies was significantly higher in CTRL−, with a remarkably higher average (312,192.6 +/− 14,443.4 s.e.) than the spore dose that was inoculated at the beginning of the experiment. CTRL+ (83,777.8 +/− 13,735.9 s.e.) and N4 (105,885.7 +/− 7294.3 s.e.) resulted as the significantly least infected groups. The *N. ceranae* abundance in the groups N4, R4 (120,366.3 +/− 13,307.1 s.e.) and N2 (137,388.9 +/− 3367.0 s.e.) was not significantly different among them. Similarly, no significantly different number of *N. ceranae* genomic copies was found between the bees fed with the least concentrated DSM patties, N2 and R2 (159,921.4 +/− 4152.3 s.e.).

### 3.4. Total ITC Gut Analysis

To investigate the fate of GSL after bee N2 and R2 intake the search of ITC presence in the gut of uninfected bees was pursued. Gut tissues were sampled at 8 dpi and analyzed for total ITC content by the quantification of the derivatization product 1,3-benzodithiole-2-thione. The latter was obtained from different ITCs, as AITC from *B. nigra* and erucin plus sulforaphane from *E. sativa* DSM. Results are shown in Table 5.

The analysis revealed the presence of ITCs in tissues extracted from bees fed on 2% DSM patties, while no ITCs were detected in the CTRL− sample, represented by bees fed on candy alone. Different quantities were found depending on gut tissues and DSM used. In particular, the quantity of total ITCs found in samples of midgut were significantly lower than total ITCs in the hindgut, both for bee fed on R2 or N2 patties. Besides, the quantity of ITC derived from R2, mainly erucin, was higher than ITC formed from N2, represented by AITC, both for samples of midgut and hindgut.

Based on the DSM consumption registered in the last 24 h before bee gut sampling, and the related GSL intake, the GSL–ITC conversion rate, calculated for the whole gut, considering a 1:1 stoichiometric ratio, was 38.8% and 11.5% for R and N, respectively.

A random sampling of bee gut tissues was also performed on infected bees from bee treatment feeding trials described above; ITCs were found in treated bees while CTRL− were free of ITC adducts (results not shown).

## 4. Discussion

This experiment aimed to contribute to the alleviation of the *N. ceranae* impact on the beekeeping industry. Despite a quasi-global distribution and severe effects as a driver of colony losses, we still miss conclusive control measures against this pathogen [65]. Artificially infected worker honey bees were reared in hoarding cages and fed different GSL-containing diets to measure: (i) palatability and GLS intake; (ii) honey bee survival; (iii) development of the *N. ceranae* infection; and (iv) GSL conversion into ITCs in the honey bee intestines. The feedings were prepared with DSMs from *B. nigra* and *E. sativa*, which were added in the proportion of 2% and 4% to commercial candy.

Both tested plants belong to the botanical family of Brassicaceae and contain bioactive compounds, mainly GSLs, which can be obtained with low-impact cultures. DSMs are by-products of the seed oil extraction that, in the specific case of the chosen plant species are particularly rich in GSLs. The hydrolysis of GSLs in *E. sativa* (GER and GRA) and *B. nigra* (SIN) DSMs produce, respectively, the ITCs erucin, sulforaphane and allyl isothiocyanate, all of which have been studied for their beneficial effects on humans [63,66,67,68,69]. However, DSMs contain other substances (proteins, residual oils, and phenolic compounds), which normally the bee assumes by feeding on the pollen with positive effects on health, learning and other cognitive functions and that may contribute to the global effect of the formulation [70,71,72]. In the case of *E. sativa* and *B. nigra* DSMs, the protein component is rich in sulfur-containing amino acids while the erucic acid, a compound recognized as toxic for the humans [73,74,75], but of unknown effects on the bees, is the most represented fatty acid in the residual oil. Nevertheless, oils from *E. sativa* and *B. nigra* showed respectively antifungal and antibacterial activity [76,77], which may complete the ITC effect. Moreover, *E. sativa* and *B. nigra* DSMs total phenolic content is in line with other DSMs from plants of the same family [37,59], albeit their composition could be highly diverse depending on agri-environmental factors [78]. Their known antioxidant properties could also enhance the nutritive and protective effect of selected DSMs on bees.

Patties added with DSMs from the two plants at the concentration of 4% showed substantial steadiness in their GSL composition within the 10-day time interval of the stability experiment. SIN concentration did not change significantly during the storage, whereas GER and GRA showed slight but significant variations. In detail, GER lost 5.5% of its initial concentration, while GRA increased it of 7.6%. This behavior is not surprising as GER could have partly undergone oxidation to GRA, anyway this partial conversion should not be considered worrisome for the final biological activity of the formulates. The deriving bioactive compounds, erucin and sulforaphane, could probably exert a similar biological activity considering they were reversible biotransformed one in the other, in the human body [79]. On the other hand, GSLs stability in formulated patties indicated that they were not hydrolyzed by MYR enzyme; thus, demonstrating a successful enzyme deactivation in DSMs. Preliminary studies showed that the presence of DSM–MYR activity led to GSL hydrolysis to ITC in patties and highly affected patties palatability (not shown).

Regarding the treatment feeding trial results, the analysis of bee cumulative individual food consumption data showed no statistical differences between *B. nigra* and *E. sativa* DSM patties. Two-per cent added feedings resulted in significantly more consumed compared to 4% DSM patties and unadded candy, allowing the hypothesis of a stimulating effect of moderate additions to a plain sugar medium. The higher consumption may have compensated to some extent the lower concentration, as no clear difference in GSL intake was detected among the treated groups. However, it should be noted that this effect is associated with a probability coefficient (*p* = 0.051) only slightly above the conventional threshold adopted to protect against Type I errors.

No significant difference in bee survival was detected between untreated and treated controls. This result is compatible with low acute fumagillin toxicity at the concentration that was used and may indicate that, within the observation period, uncontrolled infections did not reach levels affecting the honey bee survival [15,16]. Feeding 2% *B. nigra* DSM significantly increased the survival compared to both controls and 4% *E. sativa* patties. Food added with 4% *B. nigra* DSM was somewhat opposite to that, as it corresponded to a significant survival decrease compared to any other group. The hazard ratio analysis showed this concentration effect on bee survival even more clearly, as the patties added with 2 and 4% *B. nigra* DSM concentrations corresponded, respectively, to significantly longer and shorter expected time to the event of death compared to the untreated controls.

All treatments significantly reduced the development of the *N. ceranae* infections compared to the untreated controls. Fumagillin and 4% *B. nigra* DSM feeding showed ex aequo the highest inhibiting effect. Two per cent *E. sativa* DSM patties resulted the significantly least effective treatment, whereas the other DSM-containing formulations represented an intermediate statistically homogeneous group.

The results of DSM palatability, bee survival, and efficacy against *N. ceranae* highlighted that *B. nigra* DSM at the lowest dose of 2% was the best and promising treatment. Indeed, this formulation did not show a significant difference of activity against the microsporidia, but it resulted more palatable, providing the same GSL intake, and it increased beneficially the bee tolerability and survival, halving the hazard ratio respect to the higher concentration.

The analysis of gut tissues from treated bees revealed the presence of ITCs, in the form of free or adduct compounds. The cyclocondensation assay enables the quantitative detection of total ITCs, in the free form, but also as ITC-adducts (namely dithiocarbamates) possibly deriving by ITC reaction with amino acids or proteins in the medium, avoiding underestimation [80]. Since DSMs were preliminarily treated in order to deactivate MYR activity and once verified the GSL stability in the candy during trials, it can be inferred that GSL hydrolysis to ITCs occurred after DSM ingestion and derived from bee metabolism, probably by an endogenous MYR-like enzyme, normally involved in carbohydrate metabolism [81,82]. Otherwise, bee intestinal bacterial MYR-like enzymes could have contributed to GSL hydrolysis [83]. The latter hypothesis, if confirmed, could explain the higher quantity of ITCs found in the hindgut, known to host a large microbial community [84], rather than in the midgut. Instead, the higher quantity of *E. sativa* DSM derived ITCs detected in gut tissues and their apparent higher GSL-ITC conversion rate respect to AITC from *B. nigra* DSM, could be due to a higher affinity for the enzyme-substrate. Moreover, other mechanisms could be involved as a more rapid bee detoxifying strategy for AITC, or simply minor stability of AITC in the gut respect to *E. sativa* ITCs. AITC is a volatile molecule known to be very unstable in buffer or aqueous media [62]. Furthermore, preliminary investigations showed that ITCs were the major hydrolysis products (results not shown), however, it cannot be excluded that other degradation products, such as nitriles, could be formed, in the function of the medium conditions and hydrolytic enzyme biochemistry.

To the best of our knowledge, the only study reporting the use of ITCs to control honey bee disease was performed by Borges et al. against *N. ceranae* [23]. In that work, the authors demonstrated that D,L-sulforaphane, administered to bees at pure concentration dissolved in a water/sugar syrup, reduced the *N. ceranae* spore counts, but also increased the bee mortality, indicating high toxicity at higher doses in relation to high ITC concentration. In detail, a 100% *N. ceranae* spore reduction was observed after D,L-sulforaphane administration of 1.25 mg/mL in the syrup for 16 days, but 100% of bee mortality was recorded. Moreover, the real ITC intake, calculated from the cumulative feed intake reported and reformulated for 8 days (about 113 mg syrup/bee and 0.7 ¦μmol sulforaphane/bee, considering syrup density of 1.2 g/mL), seemed to be much higher respect to the GSL intake evaluated in our experiments. Instead, at the lower dose of D,L-sulforaphane (0.1667 mg/mL), which resulted in an ITC intake closer to that of GSL intake demonstrated in our assay (about 0. 16 ¦μmol sulforaphane/bee calculated for 8 days), determined a 64% reduction in the spore counts, but bee survival was again negatively affected respect to untreated control (bee mortality 23%). Nevertheless, in our study, bees were not fed with pure ITC, but with DSMs, and ITCs were evaluated as GSL metabolites in the bee gut. Furthermore, the observed DSM protective effect on bees and the control on *N. ceranae* could be linked to the ITC presence in the bee gut and their direct potential antimicrobial and/or antioxidant activities, even if other DSM components could contribute to diet beneficial effect. The ITC or GSL effects on the microsporidia spore’s reduction could be linked to their antioxidant gene promoting activities [85]. During the infection, *N. ceranae* increases the reactive oxygen species (ROS) generation and decreases the expression of antioxidant properties [13], causing higher oxidative stress that linked to the epithelial damages, allow the microsporidia to spread in new cells [11]. The ITC action, upregulating the antioxidant genes, could prevent the *N. ceranae* spreads, blocking their infection routes, even if the high expression of these genes can cause cell damage and alter the cell repair process [86].

The same correlation verified for the positive effect of ITCs could be associated with negative or toxic effect showed by *B. nigra* administration at the highest dose. Indeed, ITC antimicrobial activity is not selective to pathogens, but it could inhibit the growth of beneficial microbes too [52]. The decrease and/or alterations of gut microbes could contribute to an increase the bee mortality [23,26,27]. The ITC could induce the apoptosis of bee cells [23,52,87] and it might cause intestinal function damages, which increase bee mortality and food intake. Moreover, high ITC concentrations could have a toxic effect on bees because they down-regulate the kinases involved in the Wnt signaling pathways, reducing cell repairing, and proliferation [86]. Moreover, *N. ceranae* downregulated the gene involved in the Wnt pathway [11], and the synergic downregulation in bees treated with ITC or GSL diets could reduce the gut epithelium and their functions, increasing mortality.

## 5. Conclusions

This research shows promising results in the potential use of Brassicaceae DSMs as an integration of the bee diet. Their administration has an interesting effect on the inhibition of *N. ceranae*, and potential nutraceutical benefits involved in the bee lifespan if given at low doses. Trials in the field, at colony level, are needed to confirm the results obtained under laboratory conditions, where DSM/GSL doses should be resettled. This is the first time that ITCs were detected in honey bee gut tissues after brassica product intake. Further research is necessary to investigate the presence of MYR activity in the bee and to understand bee GSL metabolism.

## Figures and Tables

**Table 1:** Chemical characterization of *E. sativa* and *B. nigra* DSMs. Nitrogen and oil content are expressed as % *w/w* (based on dry weight). GSL content is expressed in μmol/g and total phenolic content in mg gallic acid equivalent (GAE)/g of DSM. Mean values ± standard deviation (*n* = 3) are shown. GSL common variable side chain (R) chemical structure is also indicated where X represents the GSL S-glucopyranosyl thiohydroximate moiety. GER, GRA, and SIN indicate the GSL glucoerucin, glucoraphanin, and sinigrin respectively.

	*E. sativa* DSM	*B. nigra* DSM
Moisture (%)	5.9 ± 0.1	7.6 ± 0.1
Nitrogen (%)	5.6 ± 0.1	7.0 ± 0.1
Oil (%)	17.9 ± 0.2	8.9 ± 0.1
GSL1 (¦μmol/g) side chain (R1) GSL2 (¦μmol/g) side chain (R2)	GER 72.5 ± 1.8 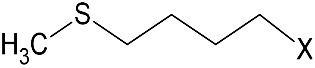	SIN 101.8 ± 2.5 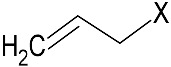
	GRA 19.7 ± 0.2 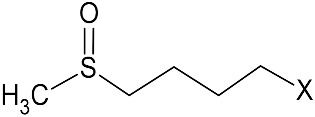	
Phenols (mg GAE/g)	8.6 ± 0.4	8.6 ± 0.2

**Table 2:** GSL content of DSM patties N4 and R4 at the beginning (T0), and the end of the trial (T10), after 10 days of storage at 33 °C. Mean values ± standard deviation (*n* = 3) are shown. GSL is expressed as ¦μmol/g of patty (±SD). Statistical differences between GSL content means are indicated by different superscript letters (*p* < 0.05, LSD test). SIN indicate the sinigrin content in N4, GER, and GRA indicate the glucoerucin and the glucoraphanin respectively content in R4.

DSM Patty	GSL	T0	T10
N4	SIN	4.07 ± 0.01	4.00 ± 0.07
R4	GER	2.90 ± 0.05 ^a^	2.74 ± 0.14 ^b^
	GRA	0.79 ± 0.03 ^b^	0.85 ± 0.08 ^a^

**Table 3:** Censored and total observation, and mean survival +/− s.e. in the treated groups (R2, R4, N2, N4), the CTRL− and CTRL+.

Group	Censured Observations	Total Observations	Survival (Days)	0.95 C.I.
CTRL−	47	75	11.125 +/− 0.511	10.124–12.127
R2	56	75	12.677 +/− 0.434	11.827–13.527
R4	52	73	11.886 +/− 0.415	11.073–12.699
N2	63	75	13.384 +/− 0.417	12.568–14.201
N4	35	74	8.798 +/− 0.508	7.801–9.794
CTRL+	53	75	11.385 +/− 0.394	10.613–12.157

**Table 4:** Between-group effects of continuous and categorical predictors included in the GLM analysis of *N. ceranae* abundance data for treated groups (R2, R4, N2, N4) and CTRL−.

Effect	Parameter +/− s.e.	t	*p*
Intercept	216,340.0 +/− 14,952.0	14.469	0.000
dpi	−7204.6 +/− 1641.4	−4.389	0.000
CTRL−	157,167.9 +/− 9409.3	16.703	0.000
R2	8551.0 +/− 8779.9	0.974	0.331
R4	−33,488.0 +/− 9029.3	−3.709	0.000
N2	−10,336.3 +/− 8475.6	−1.219	0.224
N4	−52,406.2 +/− 10,684.5	−4.905	0.000

**Table 5:** Total ITC content of midgut and hindgut tissues of uninfected bees fed on R2 and N2 for 8 days. ITC content is expressed as pmol/mg of gut tissue. Mean values ± standard deviation (*n* = 3) are shown. Statistical differences between ITC content means are indicated by different superscript letters (*p* < 0.05, LSD test).

DSM Patty	ITC Content (pmol/mg)	
	Midgut	Hindgut
R2	347.83 ^b^	788.90 ^a^
N2	21.95 ^c^	285.57 ^b^

## Data Availability

MDPI Research Data Policies.
